# Single-Stage Arthroscopic Anterior and Posterior Cruciate Ligament Repairs and Open Medial Collateral Ligament Repair for Acute Knee Dislocation

**DOI:** 10.1155/2020/7348201

**Published:** 2020-02-24

**Authors:** Ryo Murakami, Eisaburo Honda, Atsushi Fukai, Hiroki Yoshitomi, Takaki Sanada, Hiroshi Iwaso

**Affiliations:** Department of Sports Orthopedic Surgery, Kanto Rosai Hospital, 1-1 Kizukisumiyoshicho, Nakahara-ku, Kawasaki City, Kanagawa Prefecture, Japan

## Abstract

Till date, there are no clear guidelines regarding the treatment of multiple ligament knee injuries. Ligament repair is advantageous as it preserves proprioception and does not involve grafting. Many studies have reported the use of open repair and reconstruction for multiple ligament knee injuries; however, reports on arthroscopic-combined single-stage anterior cruciate ligament (ACL) and posterior cruciate ligament (PCL) repairs are scarce. In this report, we describe a case of type III knee dislocation (ACL, PCL, and medial collateral ligament (MCL) injuries) in a 43-year-old man, caused by contact while playing futsal. On the sixth day after injury, arthroscopic ACL and PCL repairs were performed with open MCL repair. The proximal lesions in the three ligaments that were injured were sutured using no. 2 strong surgical sutures. The ACL was pulled out to the lateral condyle of the femur and fixed using a suspensory fixation device. The PCL was pulled out to the medial condyle of the femur, and the MCL was pulled towards the proximal end of the femur; both were fixed using suture anchors. Early mobilization was performed, and both, clinical and imaging outcomes, were good two years after surgery.

## 1. Introduction

There is no current gold standard for treating multiple ligament knee injuries [1]. Although this type of injury is rare, accounting for less than 0.02% of orthopedic injuries [2], the instability of the knee results in persistent severe symptoms if appropriate treatment is not administered. Surgical treatment is recommended [3]; however, the specifications regarding the timing and surgical technique remain controversial. Few studies have reported on arthroscopic-combined single-stage repair [4]. Five main types of knee dislocation have been identified [5]; in this case, we treated a type III knee dislocation, as per the Schenck classification in a 43-year-old man, with arthroscopic repair of the anterior cruciate ligament (ACL), posterior cruciate ligament (PCL), and open repair of the medial collateral ligament (MCL).

## 2. Case Presentation

### 2.1. Presentation and Examination

**Figure 1:**
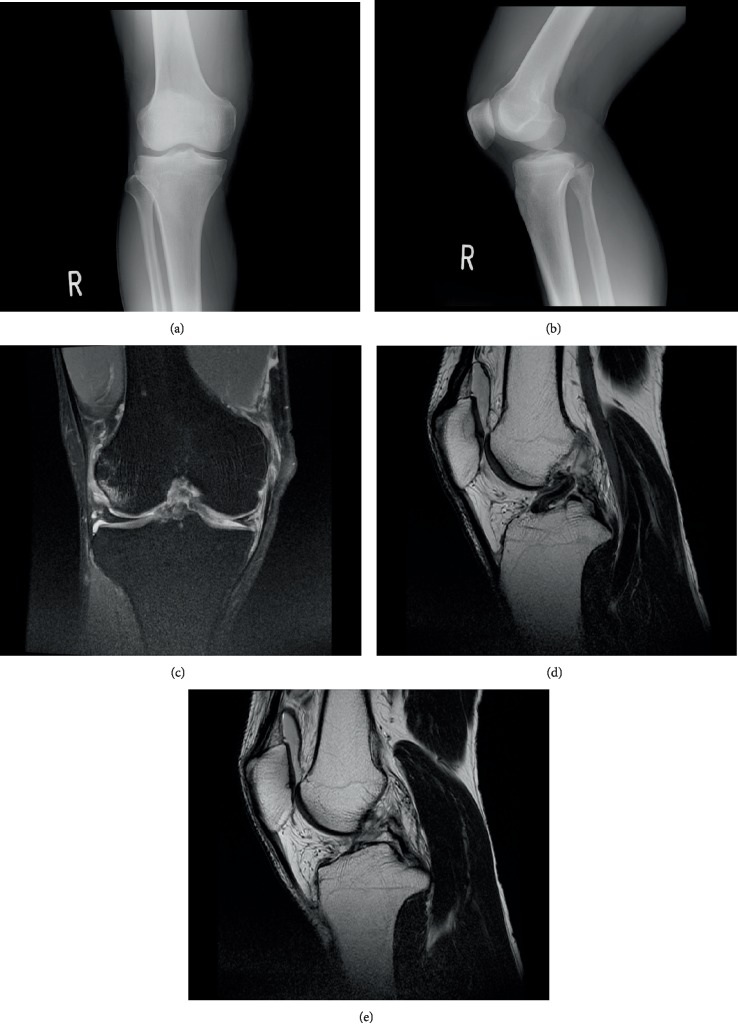
Preoperative images. (a, b) Preoperative anterior-posterior and lateral plain radiographs of the injured knee. (c) Preoperative coronal fat suppressed proton density-weighted magnetic resonance imaging (MRI) showing the proximal tear in the medial collateral ligament. (d, e) Preoperative sagittal proton density-weighted MRI showing the proximal tear in the anterior cruciate ligament (ACL) and posterior cruciate ligament (PCL).

A 43-year-old man was injured on contact while playing futsal and was diagnosed with multiple ligament injuries of the knee in an emergency hospital. During his visit to our hospital the next day, the passive range of motion (ROM) of the knee was 5-105°, and the anterior drawer test, Lachman test, pivot shift test, sagging, and posterior drawer test were all positive. The valgus stress test was found to be positive at 5°and 30° flexion. There was no patellar apprehension, and the radiographs showed no fractures; however, the magnetic resonance imaging (MRI) showed injuries to the proximal ACL, PCL, and MCL and to the medial meniscus (MM) (Figure 1).

### 2.2. Surgery

**Figure 2:**
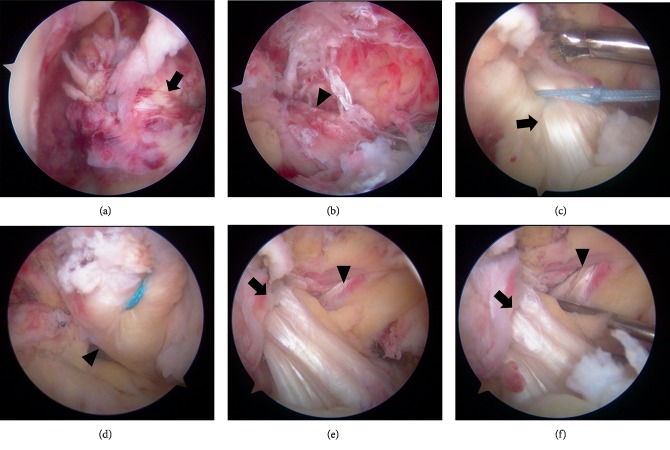
Arthroscopic images. (a, b) The ACL (black arrow) and PCL (black arrowhead) are injured proximally. (c, d) The ACL and PCL are sutured using no.2 FiberWire® by the Mason-Allen method; both were pulled out. (e, f) The ACL and PCL in tension after fixation. ACL: anterior cruciate ligament; PCL: posterior cruciate ligament.

Surgery was performed under lumbar spine anesthesia, 6 days after the injury. The patient was placed in the supine position, with the lower leg (below the knee) and feet hanging; an air tourniquet was used. Anteromedial and anterolateral portals were created, and the arthroscopic examination revealed a proximal rupture of the ACL with almost no fibers at its femoral insertion (Figure 2(a)). This injury was therefore identified as a type 1 tear of the ACL, according to the Sherman's classification [5]. The PCL was also ruptured proximally, and the stump was pinched into the medial compartment (Figure 2(b)). A horizontal tear was observed extending from the middle to the posterior end of the MM, with no cartilaginous damage.

### 2.3. Arthroscopic ACL and PCL Repairs

The severity of the ACL and PCL ruptures necessitated surgical repair; the arthroscopic PCL repair was performed first. The stump of the PCL was then pulled from the medial compartment, and the PCL was sutured using 2-0 nonabsorbable sutures. While pulling the suture with traction on the PCL, it was sutured using no. 2 FiberWire® (Arthrex, Naples, FL, USA) and TigerWire® (Arthrex), employing the Mason-Allen stitch technique (Figure 2(c)). Owing to the possibility of a considerably short distance from the stump of the PCL to the original femoral attachment, a guidewire was inserted through the anterolateral portal in a slightly lower and deeper point than the original femoral attachment of the PCL. A bone tunnel was created by drilling inside-out through the anteromedial portal, using a 4.5 mm cannulated drill. The three sutures of the PCL were pulled out to the medial end of the femur through the bone tunnel.

Similarly, the stump of the ACL was sutured using no. 2 FiberWire® and TigerWire®, employing the Mason-Allen stitch technique (Figure 2(d)). A guidewire was inserted between the original attachment site of the anteromedial and posterolateral ends of the ACL, using an outside-in guide. A bone tunnel was then created using a 4.5 mm cannulated drill, and the two sutures of the ACL were pulled out to the lateral end of the femur through the bone tunnel. The sutures of the PCL were tied to the suture of a 5.5 mm HEALICOIL (Smith&Nephew, Andover, MA, USA), inserted in the medial femoral condyle, and was fixed by applying anterior drawer force with the knee in 90° flexion. The sutures of the ACL were fixed using the Endobutton® (Smith&Nephew), with the knee fully extended.

### 2.4. Open MCL and MM Repairs

An incision was made distally from the medial femoral condyle, along the superficial medial collateral ligament (sMCL). The sartorial fascia was opened, and the ruptured sMCL and the posterior oblique ligament (POL) were identified. The ruptured sMCL and POL were then sutured using no. 2 FiberWire® in a baseball running cross-stitch pattern. The joint capsule was also found to be injured, and there was a meniscofemoral tear between it and the MM. The MM tear was sutured using 2-0 nonabsorbable sutures, and the sutures of the sMCL and POL were tied to that of the HEALICOIL and fixed at 30° flexion, with the knee in varus alignment. Both the ACL and PCL were in tension after fixation (Figures 2(e) and 2(f)).

### 2.5. Postoperative Course

Partial weight-bearing was permitted starting the day after surgery, and a ROM of 0-90° was permitted. Full weight-bearing was permitted one week after surgery, with a ROM of 0-120° being permitted 4 weeks. Six weeks after surgery, the patient was allowed a free ROM. The patient started jogging and returned to his sport at 3 and 8 months after surgery, respectively.

The ROM at 2 years after surgery was 0-140°, and there was no loss of extension or flexion compared to the contralateral knee. The anterior drawer test, Lachman test, pivot shift test, sagging, and posterior drawer test were all negative. The valgus stress test was negative at 0° flexion and positive at 30° flexion. The Lysholm, Kujala, Knee Injury and Osteoarthritis Outcome Score (KOOS) symptoms, KOOS pain, KOOS activities of daily living, KOOS sport, and KOOS quality of life scores were 99, 97, 92.8, 94.4, 100, 85, and 75, respectively. Using the KT-2000 arthrometer (MEDmetric, San Diego, CA, USA), the differences in anterior and posterior laxity between the injured and contralateral knees were +1.79 mm and +1.20 mm, respectively.

**Figure 3:**
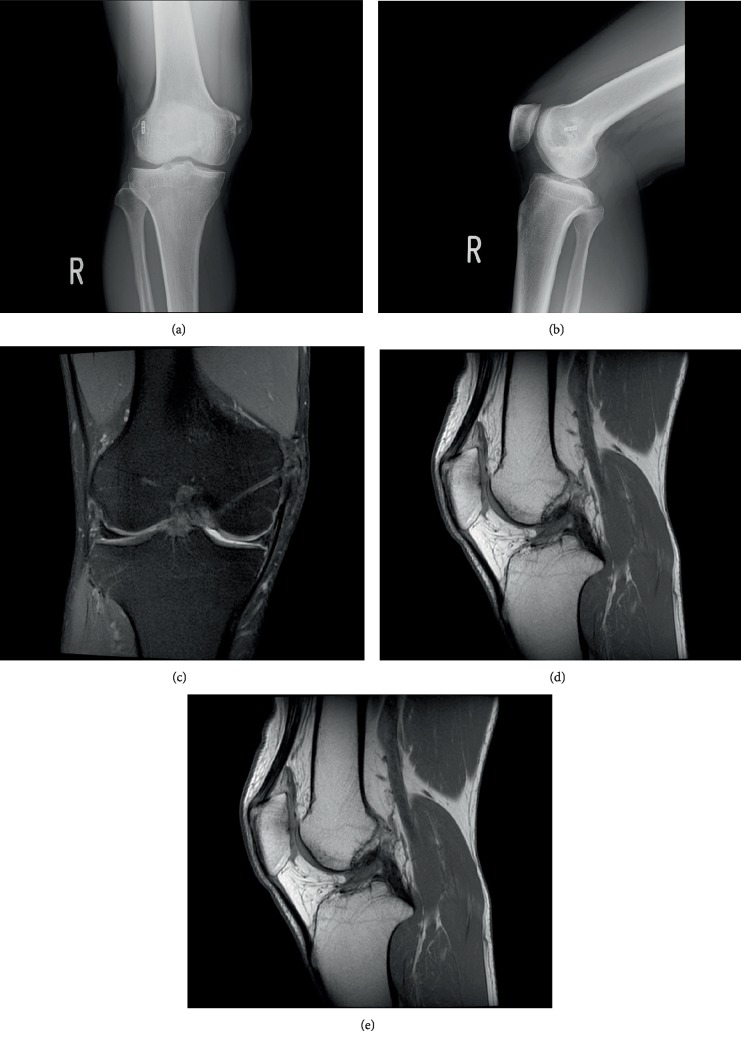
Images 2 years after surgery. (a, b) No progression of osteoarthritis seen on radiographs. (c–e) On MRI, the ACL, PCL, and MCL ligaments are in tension, and fiber continuity is maintained. ACL: anterior cruciate ligament; PCL: posterior cruciate ligament; MCL: medial collateral ligament; MRI: magnetic resonance imaging.

The radiograph demonstrated the Pellegrini-Stieda lesion but did not show progression of osteoarthritis (Figures 3(a) and 3(b)); the ACL, PCL, and MCL were in tension, and fiber continuity was maintained on MRI (Figures 3(c)–3(e)).

## 3. Discussion

Multiple ligament knee injuries are rare; however, strong knee instability remains without proper treatment, and symptoms persist. Surgery is recommended for multiple ligament knee injuries as after conservative treatment, the rate of return to sports and work, the Lysholm score, the International Knee Documentation Committee (IKDC) score, and the Lachman test results are all inferior to those observed after surgical treatment [3]. However, there are no clearly established guidelines regarding surgical methods or the timing of surgery [1].

The timing of surgery for multiple ligament knee injuries remains controversial. In a systematic review of the stage of surgery for multiple ligament knee injuries, Sheth et al. [6] reported that the Lysholm score was better in the early than the delayed surgery group, with no differences in ROM limitation. Repair or reconstruction is recommended in cases of early surgery. Additionally, in a systematic review of the stage of surgery for multiple ligament knee injuries, Levy et al. [7] reported that the early surgery group exhibited a higher Lysholm score, a higher percentage of excellent/good IKDC scores, and higher sports activity scores. However, a systematic review by Mook et al. [8] reported that anterior knee instability is more likely to persist in the early than in the delayed surgery group. In a systematic review on surgery for type III knee dislocation, Jiang et al. [9] reported that the proportions of excellent and good IKDC scores were similar for early and delayed surgeries (58.4% vs 45.5%); the proportion of 79.1% in the staged surgery group was higher than the early and delayed surgery groups. Arthrofibrosis is a complication of early surgery. Owing to a limited ROM, 45% of patients in the early multiple ligament reconstruction group required additional postoperative treatment [10]. Additional surgery is needed due to greater restriction of flexion in the early than in the delayed surgery group. In cases of early surgery for multiple ligament knee injuries, early mobilization is recommended for avoiding arthrofibrosis [8]. Although significantly more frequent manipulation was required in the early than in the delayed surgery group, there was no difference between the groups when early postoperative mobilization was performed, and functional outcomes in the early surgery group were better than those in the delayed surgery group. Therefore, both, early surgery and early postoperative mobilization, are recommended for multiple ligament knee injuries [7]. In this case, mobilization was performed the day after surgery, and there was no loss of extension or flexion 2 years later.

Controversies remain regarding the selection of repair or reconstruction as optimal treatment for multiple ligament injuries to the knee. Owens et al. [11] reported that 25 patients who underwent open multiple ligament repair for knee dislocation had a Lysholm score of 89.0 points, an extension loss of 1.9°, and a flexion loss of 10.2°4 years after surgery. Similarly, Hua et al. [12] followed up 17 patients who underwent open multiple ligament repair for an average of 4.8 years and reported that their average Lysholm score was 87.5 points; the anterior and posterior laxities were 1.4 mm and 0.8 mm, respectively. Fanelli et al. [13] reported the results of 35 patients who underwent arthroscopically assisted combined reconstruction of the ACL and PCL. In these patients, the mean postoperative Lysholm score was 91.2 points, the mean postoperative anterior side-to-side difference was 1.0 mm, and the mean postoperative posterior side-to-side difference was 2.6 mm, after more than 24 months of follow-up. Several studies have compared repair and reconstruction. Mariani et al. [14] reported no difference in functional outcomes between multiple ligament repair and multiple ligament reconstruction; however, reconstruction resulted in a lower rate of flexion loss and a higher rate of return to preinjury activity level than repair surgery. Richter et al. [2] reported that trans-osseous fixation of the ACL and PCL was superior to suturing of the ACL and PCL, and they showed no significant difference from ACL and PCL reconstruction in terms of postoperative Lysholm and Tegner scores.

Multiple ligament repair is advantageous as it utilizes native kinematics, protects proprioception, and does not involve grafting or large bone tunnels. Conversely, the disadvantage is that the adaptation is limited (i.e., damaged lesion or period after injury) [15]. Regarding indications for ligament repair, the ACL and PCL are both considered to be injured in proximal lesions [16, 17], and the MCL is considered injured in proximal or distal lesions [18]. In this case, the ACL, PCL, and MCL were also all injured proximally.

There have been certain reports of multiple ligament repair, including one case of arthroscopic repair in recent years. Kohl et al. [19] reported good results using primary ACL stabilization, with dynamic intraligamentary stabilization and PCL and collateral ligament repairs for multiple ligament knee injuries. During a mean follow-up of 2.2 years, the Lysholm score was 90.8 points, the anteroposterior laxity compared with the contralateral knee using KT-2000 was +2.5 mm, 74% of patients had a normal valgus test result in 30° flexion, and 83% of patients had a positive varus stress test in 30° flexion. There are few reports of single-stage arthroscopic suturing repair of the ACL and PCL with open collateral ligament repair. Jonkergouw et al. [3] reported on multiple ligament repair with suture augmentation. They used no. 2 strong sutures for proximal ACL and PCL tears and fixed the injury with suture anchors and distal pullout; in the proximal and distal ends, the MCL was fixed with suture anchors. However, no postoperative outcomes were reported. In ligament repairs using only the original ligament, the insufficient length of the ligament is a problem. However, in this case, the PCL bone tunnel was created at a slightly lower and deeper point than the original femoral attachment of the PCL to reduce the required length. The bone tunnel for PCL was in close proximity to the medial femoral condyle and was fixed with the suture anchor for MCL repair. Therefore, the fixation device was different for ACL and PCL in this case. The clinical and functional outcomes were good.

Arthroscopic multiple ligament repair is a challenging surgery; however, it offers certain advantages as it preserves the native kinematics and proprioception. In this case, a single-stage arthroscopic ACL and PCL repair, an open MCL repair, and early mobilization were performed for a multiple ligament knee injury. Both clinical and imaging outcomes were good, 2 years after the surgery. In cases where the injury is diagnosed by MRI and the severity of the ligament injuries are enough to warrant surgery, arthroscopic ACL and PCL repairs and open collateral ligament repair are viable treatment options for multiple injuries to the ligaments of the knee.
